# Application and prospects of single cell sequencing in tumors

**DOI:** 10.1186/s40364-021-00336-2

**Published:** 2021-12-11

**Authors:** Ruo Han Huang, Le Xin Wang, Jing He, Wen Gao

**Affiliations:** Department of Oncology, The First Affiliated Hospital of Nanjing Medical University, 300 Guangzhou Road, Nanjing, 210029 China

**Keywords:** Single cell sequencing, Tumors, Tumor microenvironment, Clinical applications

## Abstract

Cancer is an intricate disease with inherent intra-tumor heterogeneity at the cellular level because of genetic changes and environmental differences. Cellular heterogeneity exists even within the same tumor type. Small deviations in a genome or transcriptome can lead to significant differences in function. Conventional bulk population sequencing, which produces admixed populations of cells, can only provide an average expression signal for one cell population, ignoring differences between individual cells. Important advances in sequencing have been made in recent years. Single cell sequencing starts in a single cell, thereby increasing our capability to characterize intratumor heterogeneity. This technology has been used to analyze genetic variation, specific metabolic activity, and evolutionary processes in tumors, which may help us understand tumor occurrence and development and improve our understanding of the tumor microenvironment. In addition, it provides a theoretical basis for the development of clinical treatments, especially for personalized medicine. In this article, we briefly introduce Single cell sequencing technology, summarize the application of Single cell sequencing to study the tumor microenvironment, as well as its therapeutic application in different clinical procedures.

## Introduction

Malignant tumors are a common disease, and the incidence is increasing yearly. Cancer has become a considerable threat to human health [1]. The use of sequencing technology to analyze tumor genetic variation, metabolic activity, and evolutionary processes have played a major role in improving our understanding of tumor initiation and progression and has provided a theoretical basis for the development of clinical treatments. However, bulk sequencing is only helpful for obtaining average information of cells, whereas it cannot examine the heterogeneity between cells in tissues, and it is limited for studying gene expression. The reason for this is that the tissue samples used for traditional sequencing contain thousands of cells that are mixed to obtain whole genome sequence information of all cells [2]. However, cancer is not only a complex disease involving a series of pathological factors, but there is also significant heterogeneity within each tumor and between different cells [3]. Single cell sequencing (SCS) technology was developed to overcome these challenges. Single cell cDNA amplification was first reported in 1990. In 2009, Tang reported high-throughput single-cell transcriptome sequencing (scRNA-seq) [4]. In 2011, *Nature* methods listed SCS as one of the expected technologies of the year, and in 2013, *Science* magazine (Science) listed SCS as one of the six research hotspots.

At the same time, a new generation of sequencers provide powerful tools, and an increasing number of studies related to SCS have been published in top journals, indicating that SCS has gradually become a hotspot of scientific research. It is expected to become the most noteworthy sequencing technology in the future. SCS is an up-to-date technique for high-throughput sequencing analysis of genome, transcriptome, and epigenetic groups at the single cell level. Compared with bulk sequencing, SCS technology can reflect the gene structure and gene expression status of individual cells, as well as providing information on the heterogeneity between cells. More specifically, SCS can identify cell types, discover rare cell groups, reveal intratumoral heterogeneity, and define a series of cell states and evolutionary histories [5, 6]. The development of SCS technology has enabled researchers to directly examine the laws of tumorigenesis and growth, understand the causes of differences between various tumor cells and individuals, and explain the mechanism of tumorigenesis. Here, we summarize SCS technology and the existing techniques for single cell separation and amplification. We also describe the recent application of SCS to tumors and we compare the differences between sensitive and inert tumor microenvironments.

### Overview of the SCS technique

Each cell is unique. It is the core unit of structure and function. Similar to the different sized gears in a clock, cells work in close and precise coordination to maintain homeostasis in the body. SCS is accurate for exploring disease mechanisms and biological processes. Sequencing of single cells provides information on somatic mutations at the single cell level with high precision, thereby improving our understanding of the composition and interpretation of cell types in the same sample, which is widely used in cancer research. SCS mainly includes DNA-sequencing and RNA-sequencing. This technique allows analysis of the functions and features of cells at different stages and from different angles. Advances in technology have gradually improved epigenetic sequencing and single cell multi-group parallel sequencing techniques.

**Fig. 1:**
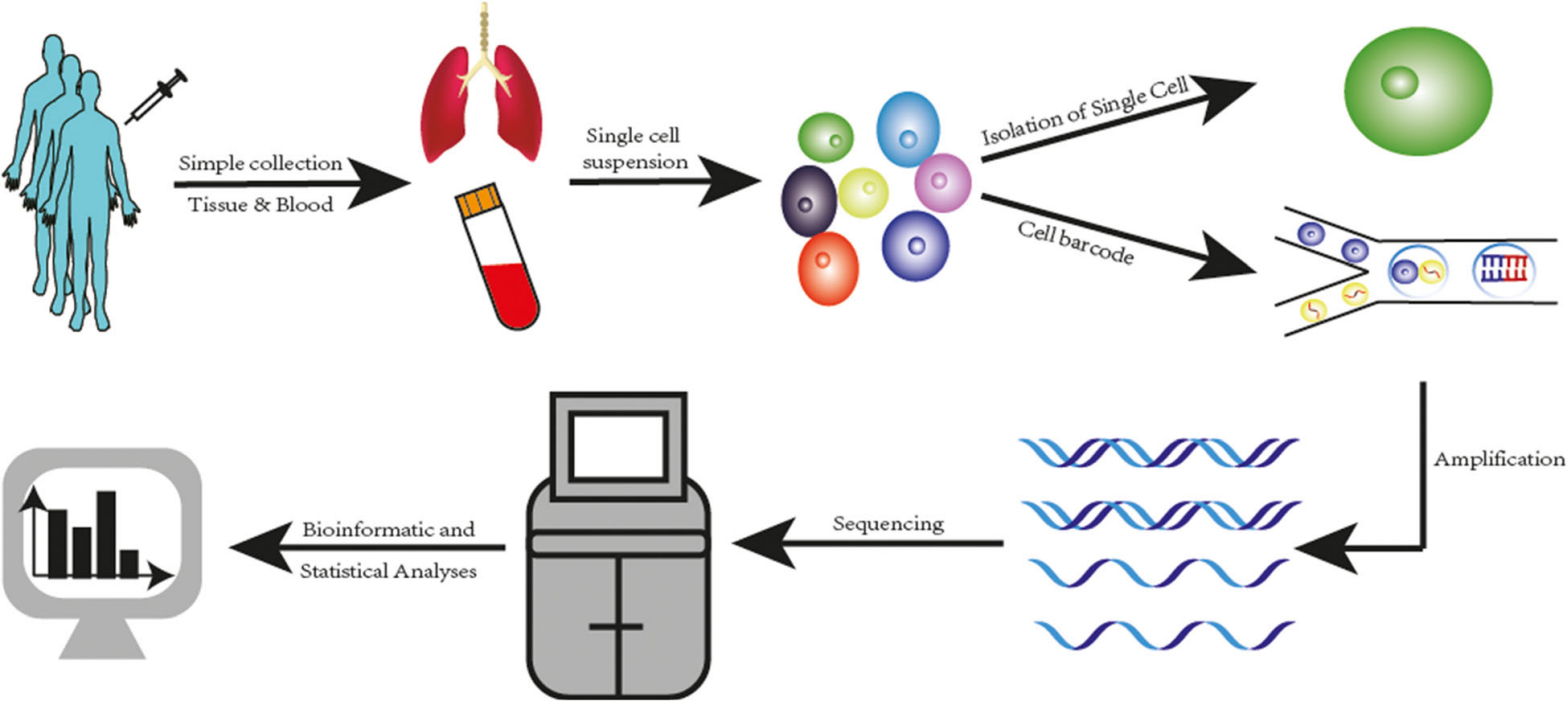
Flow chart of single-cell sequencing technology. SCS of tumor tissues can be summarized in the five steps: (1) simple collection; (2) single cells are isolated or add a unique barcode to each cell; (3) amplified; (4) and sequenced; followed by (5) bioinformatics and statistical analyses

SCS of tumor tissues can be summarized in the following steps: (1) simple collection; (2) single cells are isolated or add a unique barcode to each cell (3) amplified, (4) and sequenced; followed by (5) bioinformatics and statistical analyses [7]. (Fig. 1).

### Acquisition of single cells

The difference between population sequencing and SCS is that the latter requires isolating a single cell in good condition. There are several methods for isolating single cells. The limited dilution method is a commonly used technique in which a cell suspension is passed through a moving pipette and a liquid transfer machine for separation. Microdroplets is another popular method. It is the encapsulation of individual cells in μl-level droplets, which are piggybacked onto the enzyme used to build the library, and each microdrop contains a unique barcode [8]. Micromanipulation is the manual separation of individual cells under a microscope [9]. In flow-activated cell sorting (FACS), cells are labeled with fluorescent monoclonal antibodies that recognize specific surface markers, which enables the classification and recovery of different populations [10]. Microfluidic technologies manipulate microliter to microliter samples through micron-level flow channels [11]. Laser capture microdissection (LCML) involves identifying the target cells to be manipulated through a microscope, and the laser will excise and separate the extracted cells from the marked area according to the trajectory [12]. Here are some powerful platforms for high-efficiency or high-throughput single-cell Isolation too, such as 10x Genomics [13]. All in all, different methods can be selected according to the clinical needs. (Table 1).

**Table 1:** Main methods for isolating single cells

	Advantage	Disadvantage	Throughput	Effciency	Price	Ref
Limiting dilution	Simple	Time-consuming, easy to pollute	Low	Low	Low	[9]
Microdroplets	convenient	High cost	high	high	high	[8]
Micromanipulation	Visual, High success Rate	High technical requirements for operators, possible pollution	Low	Low	Low	[9]
FACS	Wide application、Can sort tumor cells with complex molecular markers, Technical maturity, Standard unification	Damage to cells, requaire large initial cell count	High	High	Low	[10]
Microfluidcis	High degree of automation, reduced pollutants, low sample consumption	High cost	High	High	High	[11]
LCM	Spatiality fast	Easy to destory cells, accuracy is poor	low	low	High	[12]

### Single cell DNA sequencing

In 2011, Navin invented the first single-cell nuclear DNA sequencing method for sequencing mammalian cell genomes [6]. Since then, single-cell genome sequencing technologies have flourished. Single-cell DNA sequencing can provide information on genetic heterogeneity and cell pedigree [14, 15]. However, compared with RNA sequencing, genome sequencing is challenging because each cell has many RNA molecules, whereas it has two copies of DNA. Each cell contains approximately 6 pg of genomic DNA. The genetic material extracted from a single cell is inadequate for whole genome sequencing and analysis. Therefore, single cell whole genome amplification (WGA) is necessary for single cell DNA sequencing, and ideally, the amplification procedure should have minimal deviation and sequence errors [7]. The most frequent methods are multiple displacement amplification (MDA) [16],Polymerase chain reaction (PCR) [17], or a combination of two methods of gene amplification, such as multiple annealing and looping based amplification cycles (MALBAC) [16] or Linear Amplification via Transposon Insertion (LIANTI) [14]. There are also several potential problems about WGA, such as allelic deletion (the two alleles are not amplified at the same time), low genome coverage and lack of methods to count DNA molecules, or inherent chemical instability of nucleic acids. Jay and Andrew have developed a tagmentation-based sequencing method that fragments DNA by using Tn5 transposons [18]. This method uses less DNA, but still provides a fair amount of coverage. More research efforts have provided effective solutions to some of these problems [19]. (Table 2).

**Table 2:** Technical characteristics of single cell genomic amplification methods

	Advantage	Disadvantage	Throughput	Ref
DOP-PCR	Good uniformity	A large amount of sequence information is lost, a bias in amplification, low coverage	Low	[17]
MDA	Simple, high coverage	A bias in amplification, may lead to gene fusion and allele loss.	Higher	[16]
MALBAC	Good uniformity, high accuracy, good fidelity, both fresh and fixed single-cell samples can use	Efficiency is relatively low.	High	[16]
LIANTI	High coverage, good uniformity, low error rate	High false positive rate of C-T base pairs	High	[14]

### Single cell RNA sequencing

The genotypes of cells from the same tissue are almost the same, whereas gene expression varies among different cells. This constitutes heterogeneity of gene expression, which is caused by differences in the genome, cell cycle, and microenvironment. Single cell transcriptome sequencing can dynamically represent the total RNA produced by strains or a particular cell at a certain functional stage, and is thus better for defining the cell type [20]. However, only 1–10 pg of RNA is contained in each cell, which does not meet the minimum sample requirement of the existing sequencers. Therefore, the first problem that needs to be solved for scRNA-seq is RNA amplification. The CEL-seq technique was published in *Cell Reports* in 2012 to replace PCR with in vitro transcription for amplification [21]. The MARS-seq released in 2014 is similar to CEL-seq [22]. Smart-Seq (switching mechanism at the 5′ end of the RNA transcript) is a landmark technology that can cover full length transcripts and achieve transcript isomer analysis and single nucleotide variant detection. Smart-seq2 is an improved version of smart-seq that can produce full-length transcripts and is fit for the detection of selective splicing events and allele-specific expression [23].

Advances in technology have enabled analysis of complex organs by sequencing tens of thousands of cells simultaneously; however, low cost and large-scale sequencing methods are needed. Drop-seq technology has marked a high-throughput era in single-cell transcriptome sequencing [24]. These methods can be roughly divided into two categories, full-length sequencing, represented by Smart-seq2, and label sequencing, which only captures the 3′ terminus (e.g., Drop-seq) or 5′ terminus (e.g., STRT-seq) of the sequence [23]. Compared with the methods that capture only the 3′ or 5′ end, the full-length scRNA-seq method has advantages for subtype analysis, allele expression detection, and RNA editing and identification. For detecting genes expressed at low levels, the full-length scRNA-seq method is superior to the 3′ end sequencing method. However, full-length sequencing is not suitable for high-throughput sequencing platforms and does not allow insertion of a unique molecular identifier (UMI). Tag-based sequencing can combine UMI molecules for high-throughput sequencing at low cost. However, the disadvantages include poor sensitivity for sequencing comparison and the identification of gene isomer analysis and shear events [25]. Nowadays, 10x Chromium has been generally recognized as the most commonly-used method among high-throughput methods. It is simple, convenient, integrated cell sorting, amplification and library building. In the research conducted by Ding [25], 10x Chromium detected the most UMIs and genes per cell and showed the best quality for both the number of cell types identified and the average AUCs (the area under the receiver operating characteristic curves) across cell types.

The development of scRNA-seq technology and advances in bioinformatics methods will promote biological and clinical research and provide an important theoretical basis for further understanding the heterogeneity and dynamic mechanisms of gene expression.

There are several questions that remain unanswered. For example, because of the dynamic nature of the cell transcriptome, whether the gene expression pattern of a single cell obtained by various isolation methods is equivalent to the gene expression pattern in the original environment remains unclear [7]. To solve this problem, many studies have fixed the transcriptional state of cells with aldehydes or alcohol before isolation and processing [26]. (Table 3).

**Table 3:** Technical characteristics of single-cell transcriptomic sequencing technologies

	Transcript coverage	Amplification	UMI	Advantages	Disadvantage	Ref
Tang2009	Nearly full-length	PCR	No	Sensitive, accurate	Less cell flux, expensive	[4]
Smart-seq	Full-length	PCR	No	Sequence coverage is better	Amplification of non-chain specificity	[23]
Smart-seq2	Full-length	PCR	No	Increased output, simplified steps	Less cell flux, more expensive	[23]
CEL-seq2	3′-only	IVT (In vitro-transcribed)	Yes	Reduced contamination between samples	Existence Sequence preference	[21]
Drop-seq	3′-only	PCR	Yes	Low cost, rapid library preparation, single cell high throughput, multiple possibilities	Needed microfluidic platform, low sensitivity of single cell genes	[24]
MARS-seq	3′-only	IVT	Yes	High throughput, Strictly control amplification bias	expensive	[22]
10x Chromium	Full-length	PCR	Yes	Simple and convenient, High throughput	require large initial cell count	[27–29]
Quartz-seq	Full-length	PCR	No	reduce PCR by-products、Reducing contamination of small fragments	Amplification bias	[30]

### Spatial transcriptome technologies

When we discuss gene expression patterns, there are two dimensions of the concept, one is the spatial dimension and the other is the temporal dimension. The temporal dimension can be obtained by sampling at different time points and then sequencing the single cell transcriptome, but the spatial information of tissue samples is lost in the process of applying scRNA-seq only, so spatial transcriptomics was born [31]. There are currently four main strategies, one is to use computer algorithms to simulate the spatial morphology of reconstructed tissues based on single cell transcriptome data. The second is laser microdissection combined with second-generation sequencing, but this method requires a high level of researcher skill. The third is in situ sequencing based on high-resolution images, the most classical method being smFISH. finally, there is spatial transcriptome technology based on spatial barcoding. The common techniques used for spatial transcriptome include slide-seq, LCM-seq, seqFISH, etc. 10× Genomics has introduced Visium spatial gene expression which is a high-throughput commercial technology [32]. Spatial transcriptome technologies can give us timely insights into the metastasis of tumor cells and span the molecular signature of the cancer and normal tissue boundaries [33].

### Single-cell transcriptomics combined with TCR

The TCR is a specific molecular marker of T cells that is widely used to monitor the clonality and diversity of T cells and that changes considerably under different disease conditions. By combining TCR and single cell transcriptome sequencing, it is possible to link T cell phenotypes (e.g., activation, memory, and depletion) to individual specificity and TCR clonotype [5]. The 10X Genomics platform has now developed the 10X Genomics Single Cell Immune Profiling Solution technology, which allows simultaneous high-throughput sequencing of transcriptomic gene expression and adaptive immune receptor libraries at the individual cell level. It is important to help us explore the tumor immune microenvironment, capture changes in the immune microenvironment during tumorigenesis, and find new targets for immunotherapy [34].

### Single cell epigenetics

Epigenetics refers to heritable information other than genomic DNA sequences, including DNA methylation, RNA methylation, and histone modification. Although different cells have the same DNA sequence, if the epigenetic level changes, the function of the cells changes accordingly [35]. Therefore, in addition to the genome itself, epigenetic modifications regulate gene expression, especially DNA methylation. Normal methylation can regulate cell growth and metabolism, whereas abnormal DNA methylation can induce tumor formation [36]. Common current technologies include single cell reduced-representation bisulfite sequencing (scRRBS) [37], combinatorial barcoding and targeted chromatin release (CoBATCH) [38], ChIP-seq [39] and single-cell assay for transposase-accessible chromatin (ATAC-seq) [40]. Among these methods ATAC-seq is the one with sufficiently high throughput and is therefore widely used [5].

### Single cell multi-omics sequencing

Advances in SCS technology allow obtaining information on the genome, transcriptome, and epigenome from the same cell. It is helpful to study the relationship among the three to study the process of tumor occurrence and development [41]. Current representative technologies include DR-seq, G&T-seq, scM&T-seq, and scTrio-seq [42].

### Tumor ecology

#### The tumor microenvironment

The tumor microenvironment is the internal environment in which tumor cells grow and survive. It is composed of the tumor cells themselves, as well as endothelial cells, immune cells, fibroblasts, and other cells around tumor cells. It also includes the stroma, microvessels, and biomolecules infiltrated in the adjacent area. It has an important impact on tumor growth, angiogenesis, immune escape, distant metastasis, and the response to various treatments [43]. Compared with traditional sequencing, SCS can effectively distinguish the genomes of tumor cells from those of normal cells in the microenvironment. Accurate identification of different cell groups in the microenvironment and the biomarkers that can be used to describe these cells can reveal their developmental and functional state. For example, a team developed copy number karyotyping of aneuploid tumors (CopyKAT), which can classify tumor cells and other cells according to aneuploid copy number spectrum, and even correctly analyze the genomic location of interstitial chromosome breakpoints [44]. This has improved our understanding of the tumor microenvironment [45]. Analysis at the single cell level not only describes the tumor microenvironment at an unprecedented resolution, but also allows determining how immunosuppression develops in the tumor microenvironment.

#### Immune cells in the tumor environment

Tumor-infiltrating immune cells are an important part of the tumor environment. These immune cells play a role in the occurrence and development of tumors, although our understanding of these cells remains limited. SCS technology can specifically identify certain types of cell in the tumor environment and their corresponding gene expression characteristics, thereby revealing their developmental and functional status. We list some applications of single-cell sequencing in the microenvironment by immune cell type. CD8 + T cells can kill tumor cells by secreting cytotoxic factors. Single cell level analysis revealed the presence of many exhausted CD8+ T cells expressing high levels of inhibitory receptors (IRs) in the microenvironment, such as PD-1, TIM-3 (T cell immunoglobulin-3), and LAG-3(Lymphocyte-activation gene 3). It provides an immunosuppressive environment for tumor growth [46]. Hepatoma cytotoxic CD8+ T cells continue to evolve at different stages. The rate of CD8+ T cells in early liver cancer cells is higher, showing a strong cytotoxic effect, whereas in advanced liver cancer cells, the proportion of depleted CD8+ T cells increases, the proportion of cytotoxic CD8+ T cells decreases, and the killing ability decreases [47]. The combination of scRNA-seq and scTCR-seq shows that pre-existing T cell receptors (TCRs) in tumors are different from most TCRs, indicating that CD8+ T cells are in a state of constant renewal [48]. Another combined scRNA-seq和scTCR-seq study found an increase in the number of both activated and depleted CD8+ T cells after treatment in patients with advanced basal cell carcinoma or squamous cell carcinoma treated with anti-PD1 antibodies. And depleted TIL clones present before treatment did not expand after treatment and did not transition to an undepleted phenotype [49]. CD4+ T cells also play a critical role in cancer immunology [50]. Marco et al. found that tumor-infiltrating Treg cells are upregulated in several immune checkpoints and express specific signaling molecules on the cell surface [51], such as interleukin-1 receptor 2 (IL1R2), programmed death (PD)-1 LIGAND1, PD-1 LIGAND2, and CCR8 (C-C Motif Chemokine Receptor 8) chemokines, which contributed to the immunosuppressive tumor microenvironment in non-small cell lung cancer (NSCLC) and colorectal cancer. The ST2 (Suppression of Tumorigenicity 2) gene is similarly upregulated in lung adenocarcinoma (LUAD) [52]. B cells are abundant in the tumor microenvironment, although the type of B cells present in tumor tissues and the existence of subtypes remain unclear. SCS can infer populations of B cells that cannot be detected by other analytical methods [53, 54]. In addition, tumor-infiltrating myeloid cells play an important role in tumor growth and progression. These cells are diverse and may promote or limit tumor growth. However, because TIM (Tumor-infiltrating myeloid) cells lack unique cellular markers and conservation between human and mouse models remains controversial, our understanding of this cell type is limited. SCS allows sampling the entire transcriptome of a single cell, free of predefined cell surface markers and species status [48]. TAMs (Tumor-associated macrophages) make up the majority of TIMs. SCS of immune cells in NSCLC revealed that there is a transformation process between M1 and M2 macrophages, and the upregulation of Interferon Regulatory Factor 2 (IRF2), IRF7, IRF9, and STAT2 (Signal transducer and activator of transcription 2) transcription factors may promote differentiation to M2 [55]. Other studies have shown that TAM groups can promote tumor growth [56, 57].Moreover, The advent of the spatial transcriptome has provided more specific insight into the tumor microenvironment, and investigators have found that in cutaneous squamous cell carcinoma, macrophages and Treg were found to be most abundant at the interstitial boundary of the tumor, while CD8 T cells and neutrophils were largely excluded from the tumor, suggesting that Treg localization may prevent effector lymphocytes from entering the tumor causing the presence of immunosuppression in the microenvironment [58].

#### Non-immune cells in the tumor microenvironment

Among the components of the tumor microenvironment, in addition to immune cells, non-immune cells such as fibroblasts and endothelial cells are also involved in the development of tumors. Endothelial cells are the main components of the blood vessels in the tumor and significant components of the tumor microenvironment. Activated fibroblasts near tumor cells, which are called cancer-associated fibroblasts (CAFs), are the most abundant host cell components in most tumors [59]. SCS provides the possibility to further identify non-immune cells.

One of the most important applications of SCS technology is to distinguish subtypes that have never been found in various non-immune cells [60]. In a breast cancer study, SCS technology helped researchers identify a variety of CAF subtypes, including vCAFs that originate from perivascular cells and are invasive [61]. Similar studies were performed for pancreatic ductal adenocarcinoma (PDAC) [62].

SCS can also compare the functional changes in normal tissues by analyzing the expression factors of non-immune cells in the tumor microenvironment. Tirosh et al. found that CXCL2, CCL9 ((C-C motif) ligand 9), and other chemokines expressed by CAFs, as well as the immunoregulatory genes PD-L2 and complement factor, are involved in the regulation of tumor infiltration of T cells [61]. Baryawno et al. demonstrated that fibroblasts expressing CXC chemokine ligands 2 (CXCL2) are associated with aggressive solid tumors [63].

**Fig. 2:**
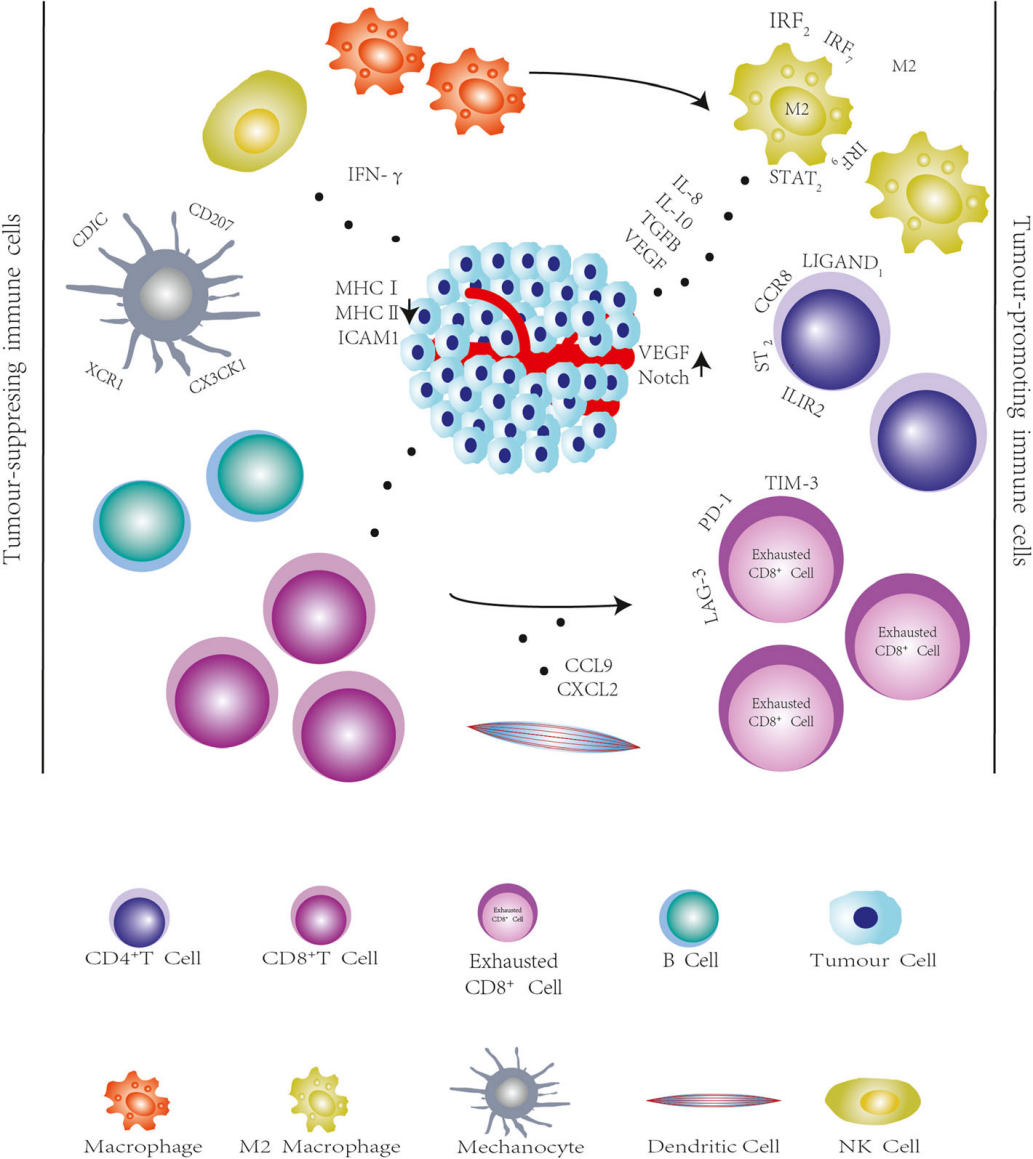
Immunosuppressive tumor microenvironment. The internal environment of tumor is a dynamic process during the development of tumor. Single cell sequencing can monitor the changes in the internal environment during the development of tumors and quantitative determination of the number of immune cells in different types of tumors

Downregulation of MHC I (Major histocompatibility complex I), MHC II, and ICAM1 (intercellular adhesion molecule) in tumor endothelial cells suggests that the antigen presentation and homing abilities of immune cells are decreased, thereby promoting tumor immune tolerance. In a study of metastatic LUAD, endothelial cells have high levels of VEGF (Vascular endothelial growth factor) and Notch signaling, indicating that tumor endothelial cells may undergo remodeling and their immune-stimulating function is inhibited, leading to tumor immune tolerance [64]. (Fig. 2).

#### Tumor evolution

Tumors are clonal diseases that result from the mutation and progressive accumulation of cellular genetic material. Single-cell sequencing technology can help us identify many abnormal genetic alterations associated with tumorigenesis and development as well as subpopulations of cells that play a key role in tumor development, driving the development of individualized therapy [65]. For example, Using ATAC-seq, the researchers identified many extrachromosomal circular DNAs in different cancer species, many with known cancer driver genes. And if eccDNA contains oncogenes, then amplification of such eccDNA in tumor cells would increase the fitness of tumor cells. Increasing our insights into the evolution of tumor cells [66]. Yang using single-cell sequencing of specimens from three bladder cancers identified six genes not previously found in bladder cancer and revealed that co-mutations in ARID1A [67], GPRC5A and MLL2 enhanced the self-following ability of bladder cancer cells. In 2011, researchers sequenced breast cancer cells, and the finally results suggest that tumor evolution may have occurred intermittently [6]. In addition, single-cell sequencing helps to reconstruct a more comprehensive and accurate tumor cell lineage tree, which in the past was often based on data from multicell sequencing and ignored certain trace genes, which can be well avoided by single-cell sequencing [68].

### Clinical application of single cell sequencing

Advances in technology have led to the increased use of SCS as a clinical guide. Here, we discuss its use in diagnosis, treatment, and prognosis prediction.

#### Single cell sequencing for diagnosis

SCS is used for the diagnosis of several diseases. Various tumor-related gene mutations are used in the clinic as biomarkers for the diagnosis of specific types of tumors [69]. SCS can identify markers related to tumor diagnosis, and it can detect copy number variation to differentiate malignant from benign cells, thereby providing a basis for early diagnosis.

Bladder cancer (BC) is a common tumor with a high incidence of relapse. A simple and convenient screening method is needed to facilitate early diagnosis. The common method is to test exfoliated tumor cells (ETCs) in the urine. However, when the number of ETCs is too small or the sample contains other types of cells, the sensitivity of this method is limited. Surveying oncogenic driver mutations (e.g., FGFR3 and TERT) or genome-wide copy number variations in ETC can be accurately detected by SCS [70]. Similarly, SCS can be applied to detect BRAF gene mutations in melanoma [71], KRAS gene mutations in colon cancer, and EGFR gene mutations in NSCLC [72, 73]. SCS provides a basis for elucidating the mechanism of cancer and helps find new markers that can be used for early diagnosis. Schlesinger et al. sequenced pancreatic cancer cells at different stages in a mouse model and found that ONECUT2 may be a driving factor for early progression [74]. Zhu et al. sequenced the tRNA derived small RNAs (tsSRNAs) of hepatic cancer patients and healthy controls and found that the plasma levels of four kinds of tsRNA (tRNA-valta-3, tRNA-glyTCC-5, tRNA-valaac-5, and tRNA-glucTC-5) were elevated in hepatic cancer patients, indicating that plasma exosome tsRNA can be used as a new diagnostic biomarker [75] . HOXA11-AS/LINC00964/MALAT1 long noncoding RNAs are used in the diagnosis of head and neck squamous cell carcinoma [76]. Although additional clinical studies are needed, we believe that SCS may play in identifying diagnostic markers.

Another application of SCS in tumor diagnosis is the combination with the liquid biopsy. Advances in sequencing technologies have allowed a large amount of molecular information to be generated in a single cancer specimen, bringing clinical oncology into the era of precision medicine, but still relying on tissue biopsy. Cancer is a spatially and temporally dynamic disease that cannot be captured by tissue biopsy. Liquid biopsy refers to the use of various body fluids, including blood, urine, pleural fluid, and other body fluids to detect circulating elements from the tumor. It allows dynamic and timely observation of changes in the tumor. One of the most studied is CTC [77]. CTCs are isolated from a primary tumor or metastatic tumor and scattered in the patient’s blood; this represents a relatively easy to obtain cancer tissue sample that can reflect the actual status of the tumor. SCS can detect CTC single nucleotide variation, CNV, or exon group insertion/deletion mutations in peripheral blood, which provides a noninvasive, highly specific, and sensitive detection method for tumor diagnosis. Some clinical trials have explored the use of CTC to assist in cancer diagnosis. For more details, see [78–81].

#### Single cell sequencing for treatment

Although there are many treatments available for cancer, efficient treatments are lacking, which is largely due to tumor heterogeneity and the dynamic evolution of tumors.

Immunotherapy has always been a popular therapy, and it is also an important part of cancer treatment. Its therapeutic principle is to overcome the immune suppression caused by the tumor and its microenvironment to allow the immune system to reactivate and kill the cancer cells [82]. However, immunotherapy is not effective for all tumors. The immune environment around the tumor can be comprehensively characterized by SCS. We found that tumor types that respond differently to immunotherapy have different immune cell compositions. Their similarities and differences can be analyzed to identify breakthroughs in immunotherapy. Table 4 lists a few simple examples. In addition, SCS can be used to analyze changes in the microenvironment before and after treatment [83]. (Table 4).

**Table 4:** The main composition of immune cells in different tumor species

	T	B	Macrophages	DC	NK	REF
Clear-cell renal cell carcinoma	+++	+	++	+	+	[109]
Breast cancer	+++	+	+	+	+	[34]
Liver cancer	+	+++	++	\	\	[110]
Nasopharyngeal carcinoma	+++	+++	+	\	+	[111]
Colorectal cancer	+++	++	\	\	\	[112]
Esophageal cancer	+++	+	++	+	+	[88]
NSCLC	+++	++	++	+	+	[113]

SCS technology enables the identification of targets for immunotherapy. Immune therapy currently focuses primarily on T cells (PD1/PD-L1, Cytotoxic T-lymphocyte-associated protein 4) or Tumor-associated macrophages (Colony-stimulating factor 1 receptor). SCS can help identify immunotherapy targets beyond these cells. One study found that CAFs can be tested in nearly all patients with advanced bladder cancer using SCS. Because CAFs secrete various tumor growth factors in the tumor microenvironment, targeting CAFs may be an ideal treatment [84]. A similar study includes pancreatic ductal carcinoma. PDAC is characterized by fibrosis and a large number of CAFs. Elimination or inhibition of CDH11 (expressed by CAFs in the pancreatic tumor stroma) can reduce the growth of pancreatic tumors and enhance their response to gemcitabine by sc-RNA [85]. Tumor infiltrating myeloid cells are another research hotspot. Macrophages are one of the main components of the tumor microenvironment. In gastric cancer, tumor cells control the anti-inflammatory properties of tumor associated macrophages, and combination therapy targeting cancer cells and macrophages could have a cooperative effect [86]. For example, M2-like macrophages induce RhoA, which regulates the migratory and invasive ability of cancer cells, and these effects can be weakened by Rho-associated protein kinase inhibitors. Therefore, blocking the Rho-GTPase RhoA is a feasible method [87].

SCS was used to sequence the infiltrating immune cells in esophageal squamous cell carcinoma, resulting in a detailed characterization of immune cells and the identification of many potential therapeutic targets. For example, macrophages express high levels of LILRB1 (Leukocyte Ig-like receptor B1), enhancing the phagocytic function of tumor cells in vivo and in vitro, and blocking this pathway may enhance the antitumor immunity of esophageal squamous cell carcinoma. NKs express high levels of checkpoint molecules such as NKG2A and CD49. Isolated blocking of NKG2A and CD49D or in combination with anti-PD1/PDL1 can improve the efficacy of immunotherapy [88]. Similarly, studies have identified a number of potential immunotherapeutic strategies for analyzing innate lymphoid cell transformation in colorectal cancer by scRNA-seq [89].

SCS technology revealed that during the process of tumor occurrence and development, the expression of some genes or proteins is upregulated or downregulated, suggesting that these genes could be therapeutic targets. Osteosarcoma is a common bone tumor with a poor prognosis. Zhou et al. sequenced 100,987 individual cells from 11 cases of osteosarcoma and showed that T-regs, CD8+ T, CD4+ T, and NK T cells express high levels of TIGIT (T cell immunoglobulin and ITIM domain) [90]. Blocking the expression of TIGIT can enhance the killing effect of primitive CD3+ T cells, as there is a high proportion TIGIT+ cells in osteosarcoma. Patients with OS may benefit from TIGIT blocking therapy. One study used scRNA-seq to depict the diversity of conventional CD4+ T cells and regulatory T cells involved in the development of lung cancer in a mouse model. The results showed that Treg-specific inhibition of ST2 signaling can improve the anti-tumor CD8+ T cell activity and reduce the tumor burden [87]. Another study found that the Laylin gene can inhibit the killing function of CD8+ T cells [91]. Kim et al. determined that Tox is an independent factor promoting tumor-infiltrating (TI) CD8+ T cell exhaustion in human cancer by analyzing sc-RNA data [92]. Because immune checkpoint inhibitors (ICIs) merely revive the stem cell-like progenitor exhausted T cells, inhibiting the process of exhaustion may increase the effect of immunotherapy. TOX promotes cell exhaustion in tumor-infiltrating CD8+ T cells by expressing IC molecules, such as PD-1, TIM-3, TIGIT, and CTLA-4. Therefore, TOX inhibition may suppress the cell differentiation process of T-cell exhaustion, thus improving the efficacy of immune checkpoint inhibitors. These findings provide ideas for the development of targeted therapy for CD8+ T cells. The emergence of a spatial transcriptome allows us to further investigate functional differences in gene expression between cancer centers and peripherals, and how signals from tumors stimulate adjacent endothelium, facilitating the search for more suitable targets [93].

The vulnerability of malignant tumors to metastasis is also a major bottleneck in the treatment process, and single-cell sequencing can help us identify and target the mechanisms of tumor metastatic progression. Using ACAT-seq, one researcher compared the differences between primary and hepatic metastatic non-small cell lung cancer and found that NFI family transcription factors were enriched in differential chromatin open sites, suggesting that NFI family transcription factors are involved in regulating tumor cell metastasis. And Nfib exhibited functions of maintaining chromatin and distal regulatory regions open and promoting neural gene expression, suggesting an important role of Nfib in promoting cancer cell proliferation and migration [94].

SCS also facilitates accurate therapy, and targeted treatments can be designed according to the molecular phenotype of each patient [7]. For example, a recent SCS program developed the maximum likelihood calculation framework MULAN (Mutability Landscape Inference), which infers mutation rates of subclones instead of individual genes. It was able to use the results obtained to test and quantify genomic interactions. This provides a theoretical basis for doctors to make individualized treatment plans in the future [95].

#### Single cell sequencing in drug resistance

Another reason for the high failure rate of cancer treatment is that tumor cells are prone to drug resistance. However, the molecular mechanism of drug resistance remains unclear [96]. SCS is an important tool to examine the mechanisms underlying drug resistance in cancer. For example, A study combining RNA-seq and ATAC-seq confirmed two mechanisms of early resistance to cetuximab in head and neck squamous cell carcinoma, TFAP2A transcription factor and epithelial mesenchymal transition [97]. Wang et al. performed SCS and showed that T cells and NK cells are the major infiltrating cells in reactive breast tumors, whereas immature myeloid cells are the main infiltrating cells in drug-resistant tumors [98]. Another study showed that in resistant cells, epithelial-mesenchymal transition and stemness genes are upregulated [99]. In endocrine resistant breast cancer, an scRNA-seq study showed that KDM5 inhibitor resistance was due to an acquired epigenetic state, and estrogen-resistant ER+ cells had high expression of KDM5 [100]. In esophageal cancer, SCS showed different keratin 19 gene expression levels between resistant and normal cell lines, suggesting inherent paclitaxel resistance in cells [101].

In summary, SCS technology can detect different cell groups in tumor samples and gain information about the typical gene expression patterns of every cell type, as well as determine the interactions between cells [102]. This provides a technical basis to identify potential therapeutic targets and explore mechanisms of tumor resistance.

#### Single cell sequencing for predicting prognosis

Accurate evaluation of the prognosis of cancer patients is important. First, there are many cancer treatment methods, and accurate prognostic markers are necessary to determine the efficacy of treatments and for doctors to adjust the treatment plan. Second, accurate prognostic indicators can be useful for analyzing the occurrence and metastasis of cancer and to promote the development of new treatment plans. Third, knowledge of the prognosis can help patients and their families prepare psychologically for follow-up treatment in time. Below are several examples of the application of SCS to the prognosis of tumors.

According to the global cancer statistics released by the International Agency for Research on Cancer in December 2020, breast cancer has surpassed lung cancer to become the most common type of cancer in the world. Finding accurate prognostic markers for breast cancer would be a big step forward in curing breast cancer and reducing the waste of medical resources. The γδ T cells are a subtype of T cells. A study used scRNA-seq of γδ T cells from human blood and breast tumor samples. They identified five subsets of human blood γδ T cells, and three subtypes were identified in human breast cancer samples. Two of the three types of T cells found in breast tumors have corresponding subtypes in the blood. The other subtype is the only one related with better overall survival in a large cohort of breast cancer patients characterized by TCGA (The Cancer Genome Atlas) consortium [103]. Another study applied SCS technology to identify various cell types present in the normal breast. The outcome indicated that there were 10 cell types in normal breast tissue. Comparison of the gene signature of each cell type with the breast tumor gene expression profile in TCGA data set indicated that the Cluster 9 (EPCAM, * KRT6B, KRT15, KRT16, KRT81, KRT23) cell type was remarkably linked with poor prognosis in triple negative breast cancer. These cells or genes may become potential prognostic biomarkers for the survival of patients with breast cancer [104].

Lung cancer currently ranks second in incidence in the world. Chen et al. detected 159,219 cells from LUAD patients based on scRNA-seq [105]. They found that 57 genes were only detected in cancer cells, of which 51 were upregulated and six were downregulated. The expression of some of these genes was associated with prognosis. Analysis of TCGA and Gene Expression Omnibus (GEO) databases showed that high expression levels of HMGA1 and EMC6 were associated with poor prognosis. Another study found that the expression of the MHC-II gene was heterogeneous in LUAD by scRNA-seq, and high expression of MHC-II was associated with good prognosis [106].

SCS shows that the number and nature of immune cells in the tumor environment is closely related to the prognosis of the tumor. A study performed in-depth scRNA-seq of 12,346 T cells from 14 untreated patients with NSCLC. The study found two CD8+ T cell groups (CD8-C4-GZMK and CD8-C5-ZNF683). The position of CD8-C4-GZMK and CD8-C5-ZNF683 is more central than that of CD8-C6-LAYN in the one-cycle trajectory, and their fatigue score is lower than that of CD8-C6-LAYN, suggesting that they may be in the state of “pre-exhaustion”. Analysis of a LUAD cohort from TCGA showed that the higher the ratio of these two kinds of cells, the better the prognosis [107]. SCS of immune cells from hepatocellular carcinoma confirmed the existence of a subgroup of CD3 + CD8+ T cells. This subgroup of T cells secretes a large amount of XCL1, which participated in antigen presentation and attracts CD8+ T cells to exert cytotoxic effects. Patients with higher cell density had a better prognosis [47]. Recent scRNA-seq studies showed that TAMs in malignant ascites of gastric cancer had a strong M2-like phenotype, which was related to the poor prognosis of gastric cancer. Therefore, the prognosis of advanced gastric cancer can be predicted by real-time monitoring of TAMs in cancerous ascites [86].

The heterogeneity of tumors is closely related to poor prognosis. Wang et al. analyzed the single cell transcriptome map of peritoneal carcinomatosis (PC) in 15 patients with gastric adenocarcinoma [108]. According to sequencing results, PC samples were divided into two types: the gastric type dominated by gastric cells and the mixed type of gastric cells and colorectal-like cells. The prognosis of the gastric type was poor Although clinical proof is needed, we believe that SCS has a promising future in predicting tumor prognosis. (Table 5).

**Table 5:** Findings obtained from single cell sequencing

Cancer type	Key findings	Ref
Bladder cancer	Accurately detect genetic mutations or copy number changes in exfoliated urine cell. ICAF can be detected in patients with advanced bladder cancer.	[70] [84]
Pancreatic cancer	ONECUT2 may be a driving factor for early progression; CAFs expressing CDH11 promote the growth of pancreatic tumors.	[74] [85]
Gastric cancer	M2-like macrophages induce RhoA, which regulates the metastasis and invasion of cancer cells. M2-like phenotype of TAMs is related to poor prognosis in gastric cancer. The prognosis of the gastric type is worse than that of the mixed type.	[86] [87] [88]
Esophageal squamous cell carcinoma	Macrophages express high levels of LILRB1, enhancing the phagocytic function of tumor cells; NKs express high levels of checkpoint molecules, such as NKG2A and CD49d. KRT19 expression is related to drug resistance.	[88]
Osteosarcoma	Increased expression of TIGIT enhances the killing effect of primitive CD3 + T cells and high proportion of TIGIT+ cells are present in osteosarcoma.	[90]
Lung cancer	inhibition of ST2 signaling can improve anti-tumor CD8 + T cell activity and reduce tumor burden. In TCGA and GEO databases, high expression levels of HMGA1 and EMC6 are associated with poor prognosis. High expression of MHCII is associated with good prognosis. Based on CD8+ T cell phenotypes, a higher ratio of pre-exhaustion cells is associated with better prognosis.	[87] [105] [106] [107]
Liver Cancer	Plasma exosome TSRNA can be used as a new diagnostic biomarker. Laylin gene can inhibit the killing function of CD8 + T cells	[75] [91]
Melanoma	TOX promotes cell exhaustion in tumor-infiltrating CD8 + T cells by expressing IC molecules.	[92]
Breast cancer	One subtype of γδ T was associated with better overall survival. A cluster of cells characterized by EpCAM, * KRT6b, KRT15, KRT16,KRT81, and KRT23 is associated with a better prognosis. Infiltration of immature myeloid cells is associated with tumor drug resistance. In resistant cells, EMT and stemness genes are upregulated. High expression of KDM5 and ITH in estrogen-resistant ER+ cells.	[98] [100] [103] [104]

## Conclusion

**Fig. 3:**
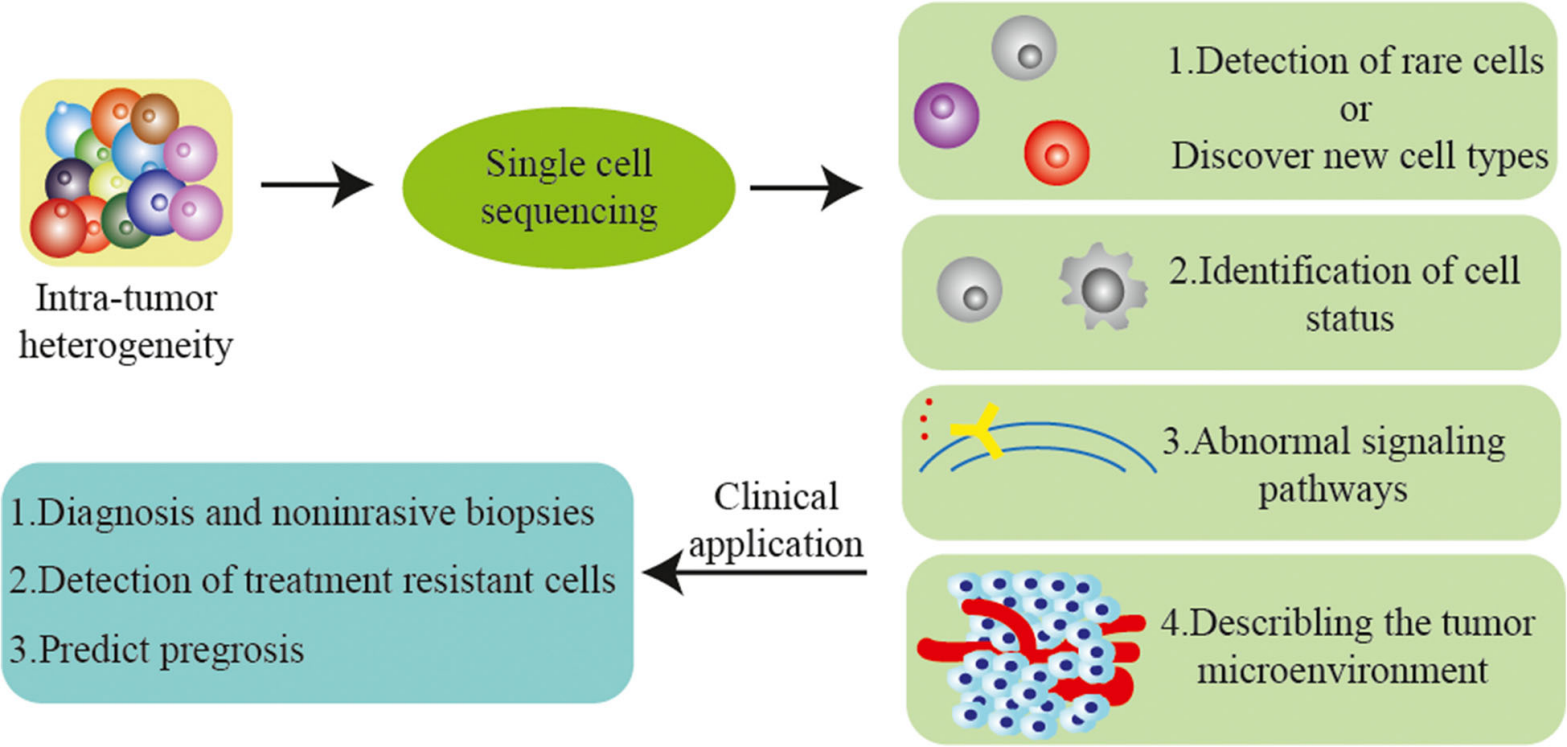
Application of single-cell sequencing in cancer research

In this review, we summarized the major SCS types and the application of SCS in the field of oncology. SCS deepens our understanding of tumors and promotes the progress of oncology. However, with the opportunity comes the challenge. There are many problems associated with SCS technology. Some issues include that it is time-consuming, associated with a high cost, the potential for technical error, and the high sample requirements, in addition to the loss of spatial structure and that only a small section of tissue can be sequenced. We believe that with the development of technology, these problems will be solved. Overall, SCS technology provides a better understanding of the tumor as a whole from the perspective of a single cell. We believe that with continuous re-optimization, SCS technology will continue to promote the development of oncology and improve the tumor diagnosis and treatment systems to pave the way for individualized treatment of tumors. (Fig. 3).

## Data Availability

Not applicable.

## Abbreviations

**SCS**: Single cell sequencing
**scRNA-seq**: single-cell transcriptome sequencing
**FACS**: flow-activated cell sorting
**LCML**: Laser capture microdissection
**WGA**: whole genome amplification
**MDA**: multiple displacement amplification
**MALBAC**: multiple annealing and looping-based amplification cycles
**PCR**: polymerase chain reaction
**LIANTI**: Linear Amplification via Transposon Insertion
**UMI**: unique molecular identifier
**scRRBS**: single cell reduced-representation bisulfite sequencing
**CopyKAT**: copy number karyotyping of aneuploid tumors
**IRs**: inhibitory receptors
**TCRs**: T cell receptors
**IL1R2**: interleukin-1 receptor 2
**NSCLC**: non-small cell lung cancer
**LUAD**: lung adenocarcinoma
**TIM**: tumor-infiltrating myeloid cell
**TAM**: Tumor-associated macrophage
**CAFs**: cancer-associated fibroblasts
**PDAC**: pancreatic ductal adenocarcinoma
**MHC**: major histocompatibility complex
**ICAM**: intercellular adhesion molecule
**VEGF**: vascular endothelial growth factor
**IVT**: In vitro-transcribed
**PD**: programmed death
**CNV**: copy number variation
**BC**: Bladder cancer
**ETCs**: exfoliated tumor cells
**tsSRNAs**: tRNA derived small RNAs
**CTC**: circulating tumor cell
**TI**: tumor-infiltrating
**ICIs**: immune checkpoint inhibitors
**MULAN**: Mutability Landscape Inference
**TCGA**: The Cancer Genome Atlas
**GEO**: Gene Expression Omnibus
**PC**: peritoneal carcinomatosis
